# The severity of intrahepatic cholestasis of pregnancy and its association with pregnancy complications and neonatal asphyxia: A single-center case analysis and systematic review

**DOI:** 10.17305/bb.2024.10588

**Published:** 2024-12-01

**Authors:** Siming Xin, Mengjiao Liu, Hua Lai, Liju Nie, Ying Hong, Yin Xiong, Xianxian Liu, Ting Wu, Xiaoming Zeng, Fen Fu

**Affiliations:** 1 Department of Obstetrics and Gynecology, The Second Affiliated Hospital, Jiangxi Medical College, Nanchang University, Nanchang, China; 2 Department of Obstetrics, Jiangxi Maternal and Child Health Hospital, Nanchang, China; 3 School of Public Health, Nanchang University, Nanchang, China; 4 Key Laboratory of Women’s Reproductive Health of Jiangxi Province, Jiangxi Maternal and Child Health Hospital, Nanchang, China

**Keywords:** Intrahepatic cholestasis of pregnancy (ICP), biochemical markers, adverse pregnancy outcomes, neonatal asphyxia, systematic review, meta-analysis

## Abstract

Intrahepatic cholestasis of pregnancy (ICP) poses significant risks to maternal and neonatal health. Our study at Jiangxi Provincial Maternal and Child Health Hospital analyzed clinical and biochemical markers in singleton pregnancies diagnosed with ICP from October 2016 to December 2022. This research, supported by a systematic review and meta-analysis of existing studies, highlights the increasing incidence of ICP and its association with elevated levels of total bile acids (TBA), transaminases, and bilirubin. Our findings indicate a marked increase in the risk of preterm birth, cesarean delivery, and neonatal asphyxia as the severity of ICP escalates. This underscores the need for vigilant monitoring and management of affected pregnancies. By confirming the relationship between biochemical marker abnormalities and adverse pregnancy outcomes, our study advocates for enhanced clinical strategies and paves the way for future research aimed at improving prevention, diagnosis, and treatment methods for ICP.

## Introduction

Intrahepatic cholestasis of pregnancy (ICP), a specific liver disorder predominantly occurring in the late stages of pregnancy, is characterized by unexplained pruritus and elevated serum total bile acids (sTBA) [1–3]. Studies highlight significant global variations in the incidence of ICP, ranging from 0.3% to 27.6% [4–7], underscoring the complexity of its pathogenesis and its close association with geographic location, ethnicity, and lifestyle habits. In China, the incidence of ICP also shows substantial regional differences due to the country’s geographical complexity, ethnic diversity, and dietary habits [8–10]. Although ICP is generally benign for the pregnant woman, it can pose severe risks to the perinatal child, including preterm birth, fetal hypoxia, and even stillbirth [9, 11–13]. Notably, research indicates that the risk of adverse pregnancy outcomes significantly increases with maternal sTBA levels [14–17].

While there is a wealth of research on ICP and its association with pregnancy outcomes, the majority of these studies have focused on pregnancy outcomes and pharmacological treatments, with less attention given to factors influencing adverse perinatal outcomes. Additionally, these studies often struggle with small sample sizes and a lack of regional data [9, 11, 18, 19]. The limitations of existing research underscore the necessity for a comprehensive understanding of the impacts of ICP on pregnancy outcomes, particularly in specific regions like Jiangxi Province. The Jiangxi Maternal and Child Health Hospital, the largest perinatal medical center in the province, receives patients from most parts of the region. Therefore, its experience in the treatment and management of ICP reveals the clinical characteristics of ICP in Jiangxi Province and reflects the actual incidence, characteristics, and trends of ICP in this area.

Given this context, it becomes essential to delve into the changes in biochemical markers associated with ICP and their relationship with pregnancy outcomes. Current research is insufficient to fully elucidate the exact relationship between biochemical marker abnormalities in ICP patients and adverse pregnancy outcomes, especially against the backdrop of the increasing trend of ICP patients and the severity of adverse pregnancy outcomes. The relationship between biochemical markers (such as total bile acids (TBA), transaminases, and bilirubin) and pregnancy outcomes (including preterm birth, cesarean delivery, and neonatal asphyxia) in patients with varying degrees of ICP severity has not been adequately studied.

**Figure 1.**
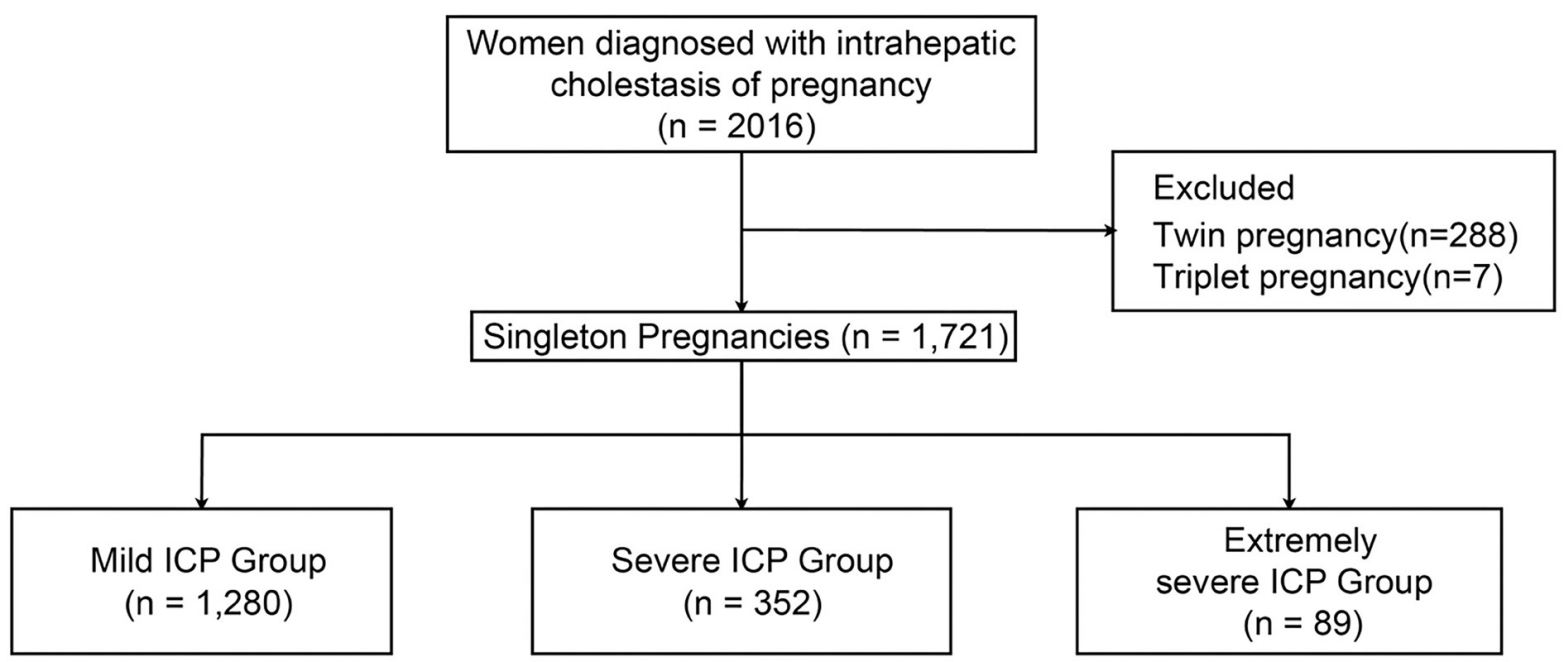
**Patient inclusion and exclusion flowchart.** ICP: Intrahepatic cholestasis of pregnancy.

Therefore, this study aims to comprehensively assess the changes in biochemical markers in ICP patients and their correlation with pregnancy outcomes through a single-center retrospective case analysis and meta-analysis conducted in Jiangxi Provincial Maternal and Child Health Hospital. We explore the clinical features of ICP, analyze the relationship between biochemical markers and pregnancy outcomes, and evaluate the correlation between the severity of ICP and pregnancy complications as well as perinatal outcomes. This study aims to fill the gaps in existing research, providing scientific guidance for the clinical management of ICP to reduce the incidence of adverse pregnancy outcomes. Additionally, it aims to offer foundational data for future research on ICP prevention, diagnosis, and treatment strategies, thereby further enhancing the health levels of pregnant women and newborns. Through this research, we hope to improve understanding of the impact of ICP on pregnancy outcomes, optimize clinical management strategies for ICP patients, reduce pregnancy complications, and improve perinatal outcomes.

## Materials and methods

### Study participants

This study is a single-center retrospective case analysis conducted from October 1, 2016, to December 31, 2022. All case data were sourced from the Jiangxi Maternal and Child Health Hospital, Jiangxi, China, retrieved through the hospital’s electronic medical record system using ICD-10-CM codes. The inclusion criteria were women from Jiangxi with singleton pregnancies diagnosed with ICP. Exclusion criteria included patients with concurrent active hepatitis, fetal anomalies, and those lacking complete medical records (Figure 1). During the period from October 2016 to December 2022, our institution recorded a total of 134,080 deliveries, with 2016 cases of diagnosed ICP among pregnant women. Among these cases, 1721 singletons met the inclusion criteria, while 288 cases of twin pregnancies and seven cases of triplet pregnancies were excluded. This study specifically focused on collecting data from singleton pregnancy cases due to the physiological and clinical differences observed between singleton and multiple pregnancies. Including data from multiple gestations could introduce additional confounding factors into the analysis. To ensure consistency and comparability of the study results, only singleton pregnancies were considered, thus enhancing the credibility of the findings. Furthermore, multiple gestations may entail a higher risk of complications, such as preterm birth and bleeding, which could potentially confound the independent assessment of ICP effects. The study adhered to the principles of the Declaration of Helsinki and was approved by the Ethics Committee of Jiangxi Maternal and Child Health Hospital (approval number EC-KT-202206, date of registration January 5, 2022).

Following the Chinese Clinical Guidelines for ICP (2015) [20], ICP was diagnosed after excluding other causes of pruritus and liver dysfunction, based on fasting sTBA concentrations ≥10 µmol/L and normalization of biochemical markers postpartum. Depending on fasting sTBA peak levels, ICP was categorized as mild ICP (TBA concentrations ≥ 10–39 µmol/L), moderate ICP (TBA concentrations ≥ 40–99 µmol/L), and severe ICP (TBA concentrations ≥100 µmol/L). Quantification of sTBA, aspartate transaminase (AST), alanine transaminase (ALT), total bilirubin (TBIL), direct bilirubin (DBIL), and indirect bilirubin (IBIL) was performed using the AU5800 automatic biochemical analyzer (Beckman Coulter) with radioimmunoassay.

The clinical baseline characteristics of all study subjects, including age, birthplace, ethnicity, height, and weight, as well as reproductive history (number of pregnancies, deliveries, method of conception), current medical history (gestational week of ICP diagnosis, skin itching, and medications during pregnancy), and biochemical testing parameters [AST, ALT, TBIL, DBIL, IBIL, and sTBA], pregnancy complications, and perinatal outcomes were documented. Pregnancy complications encompass hypertensive disorders of pregnancy (HDP), gestational diabetes mellitus (GDM), thyroid dysfunction, premature rupture of membranes (PROM), and viral hepatitis. Perinatal outcomes primarily included gestational age at delivery, mode of delivery, meconium-stained amniotic fluid (MSAF), stillbirth, birth weight, 1-min Apgar score, and 5-min Apgar score. Adverse perinatal outcomes included preterm birth, cesarean delivery, fetal growth restriction (FGR), stillbirth, and neonatal asphyxia. All pregnancy complications and delivery outcomes were derived from clinical records diagnosed by obstetricians and neonatologists at the hospital by relevant Chinese guidelines [8, 21].

### Meta-analysis literature search strategy

To thoroughly investigate and evaluate the clinical features of ICP, this study developed a detailed literature search strategy. This strategy aimed to systematically gather and analyze ICP studies, specifically focusing on the relationship between TBA, transaminases, and bilirubin levels and ICP.

The literature search was conducted across major medical databases, including PubMed, Web of Science, and CNKI (China National Knowledge Infrastructure), covering all relevant literature from the databases’ inception up to March 2024. Keywords such as “Intrahepatic Cholestasis of Pregnancy,” “ICP,” “Total Bile Acids,” “TBA,” “Transaminases,” and “Bilirubin,” and their Chinese equivalents were used. These keywords and their combinations, alongside Boolean operators (AND, OR), were utilized to construct search expressions, ensuring the precision and comprehensiveness of the search process.

To ensure the breadth and depth of the literature search, manual searches of reference lists from relevant journals and records of significant conferences were conducted, in addition to searches in the electronic databases mentioned above. This step was designed to capture critical studies that may have been missed in electronic database searches, ensuring that this meta-analysis’s data foundation is comprehensive and balanced.

### Inclusion and exclusion criteria for literature

To ensure the quality and specificity of this systematic review and meta-analysis, stringent criteria for the inclusion and exclusion of studies were employed to select literature meeting specific requirements meticulously. Study types primarily encompass randomized controlled trials (RCTs), prospective or retrospective cohort studies, and case-control studies, which must provide objective and reliable outcome measures.

Regarding the exclusion criteria, studies not meeting the standards above were eliminated, including non-human studies, commentaries, reviews, case reports, and non-empirical studies such as expert opinions. Studies involving pregnant women under the age of 18, as well as those investigating ICP caused by known factors like genetic disorders, were also excluded. Additionally, studies with incomplete data or poor methodological quality, as well as those for which the full text is not accessible or critical information is missing in the complete text, were disregarded. To avoid redundancy and bias, duplicate studies or multiple publications from the same dataset were excluded.

### Literature coding and quality assessment

For each selected study, detailed coding was carried out to record essential information including but not limited to the authors’ names, publication year, study design type, sample size, and specific data related to ICP, such as levels of TBA, transaminases, bilirubin, and gestational age. These data were organized and documented in an Excel spreadsheet to facilitate subsequent data analysis and management. The quality assessment of the literature was conducted based on the criteria set by the Cochrane risk of bias tool. This process aims to identify and mitigate potential bias risks, ensuring the accuracy and reliability of the research findings.

### Data extraction for literature

In the meta-analysis phase of this study, data extraction focused on collecting detailed information on the changes in TBA, transaminases, and bilirubin levels from each study. Additionally, each study’s sample size, experimental group configurations, and statistical significance measures (such as *P* values and confidence intervals [CIs]) were meticulously recorded. This step is crucial for analyzing the relationship between TBA, transaminases, bilirubin levels, gestational age, and ICP and assessing the reliability and statistical significance of each study’s results. By employing this systematic and detailed approach to literature data extraction, we aim to ensure the high accuracy and reliability of the meta-analysis outcomes, providing a solid scientific basis for improving the clinical management of ICP.

### Statistical methods and analysis process

Data were analyzed using IBM SPSS Statistics 24.0 (IBM-SPSS, Chicago, USA). Quantitative data were expressed as mean ± standard deviation or median with interquartile range, and comparisons between groups were conducted using chi-square tests or Kruskal–Wallis tests; qualitative data were presented as frequencies and percentages, with group comparisons performed via chi-square tests. Logistic regression analysis was utilized to explore the relationship between different severities of ICP and adverse newborn outcomes, adjusting for confounding factors, such as mother's age, number of pregnancies, number of deliveries, previous history of ICP, BMI, HDP, GDM, PROM, and viral hepatitis, to comprehensively analyze the association between adverse perinatal outcomes and clinical features of the disease.

In the meta-analysis, data analysis was conducted using R software, selecting effect size (*r*-value) and its 95% CI as indicators of effect magnitude. To assess heterogeneity among studies, Cochran’s *Q* test and the *I*^2^ statistic were applied; a random-effects model was used if *I*^2^ exceeded 50%, whereas a fixed-effects model was adopted for *I*^2^ values below 50%.

To ensure the stability of conclusions, sensitivity analyses were performed by excluding specific studies or changing analytical methods to test the robustness of the results. Funnel plots were drawn to assess potential publication bias, and, where possible, trim-and-fill methods were employed to identify and adjust for asymmetries due to loading bias [22]. The significance level for all statistical tests was set at *P* < 0.05 to ensure the statistical significance of the findings.

### Ethical statement

The present study followed the tenets of the Helsinki Declaration. Ethics approval was granted by the Institutional Review Board of Jiangxi Maternal and Child Health Hospital in China (registration number EC-KT-202206, registered on January 5, 2022).

## Results

### Analysis of incidence and severity of ICP during singleton pregnancies

By categorizing the severity of ICP cases, it was found that the majority were mild ICP, accounting for 74.38% of the total cases, while moderate and severe cases accounted for 20.45% and 5.17%, respectively (Table 1). It is notable that the proportion of mild ICP cases remained stable over the analysis period, while moderate and severe cases fluctuated without a clear upward or downward trend. Further analysis revealed an increase in the incidence of ICP from 0.98% in 2016 to 1.46% in 2022, indicating a non-random change (Table 1).

**Table 1:** Annual trends in the prevalence and severity of ICP in singleton pregnancies: 2016–2022

**Time**	**Total deliveries**	**ICP deliveries**	**Mild ICP *N* ═ 1280**	**Moderate ICP *N* ═ 352**	**Severe ICP *N* ═ 89**
Overall	134,080	1721 (1.28%)	1280 (74.38%)	352 (20.45%)	89 (5.17%)
2016.10–2017.12	29,225	285 (0.98%)	188 (65.96%)	82 (28.77%)	15 (5.26%)
2018.01–2018.12	21,946	260 (1.18%)	173 (66.54%)	69 (26.54%)	18 (6.92%)
2019.01–2019.12	22,407	320 (1.43%)	235 (73.44%)	71 (22.19%)	14 (4.38%)
2020.01–2020.12	19,971	266 (1.33%)	212 (79.70%)	39 (14.66%)	15 (5.64%)
2021.01–2021.12	20,291	294 (1.45%)	236 (80.27%)	44 (14.97%)	14 (4.76%)
2022.01–2022.12	20,240	296 (1.46%)	236 (79.73%)	47 (15.88%)	13 (4.39%)
*P* value		<0.001	<0.001		

ICP: Intrahepatic cholestasis of pregnancy.

### Association analysis between the severity of ICP and clinical features and pregnancy complications

The clinical characteristics of patients with ICP at varying severity levels indicate that there is a statistically significant difference in BMI among different grades of ICP (*P* ═ 0.004), suggesting a potential association between BMI and the severity of ICP. Differences in the distribution of previous ICP history, including ICP history and history of stillbirth, were observed among patients with varying degrees of ICP severity, with *P* values of 0.039 and 0.015, respectively, indicating a potential link between these historical factors and the severity of ICP. Moreover, as the severity of ICP increased, the gestational age at ICP onset was earlier (*P* < 0.001), suggesting that more severe cases of ICP may affect pregnant women earlier. The analysis of differences in biochemical markers (TBA, AST, ALT, TBIL, and DBIL) and the clinical symptom of pruritus reached highly significant levels (*P* < 0.001), strongly indicating that the severity of ICP is closely related to these biochemical changes and symptoms. Additionally, the incidence rates of pregnancy complications (GDM and PROM) also showed significant differences among different ICP severities, with *P* values of 0.023 and 0.020, respectively, further suggesting a potential association between these complications and the severity of ICP (Table 2).

**Table 2:** Clinical characteristics of pregnant women with ICP by severity: a detailed analysis

	**ICP**	**Mild ICP**	**Moderate ICP**	**Severe ICP**	
**Characteristics**	***N* ═ 1721**	***N* ═ 1280**	***N* ═ 352**	***N* ═ 89**	***P* value**
Age (years)	29.00 (26.00, 32.00)	29.00 (26.00, 32.00)	29.00 (26.00, 32.75)	29.00 (26.00, 33.00)	0.533
BMI (kg/m^2^)	25.84 (23.88, 28.13)	25.96 (24.00, 28.23)	25.78 (23.63, 27.69)	25.20 (23.08, 26.81)	0.004
Gravidity (times)	2.00 (1.00, 3.00)	2.00 (1.00, 3.00)	2.00 (1.00, 3.00)	2.00 (1.00, 4.00)	0.403
Parity (times)	1.00 (1.00, 2.00)	1.00 (1.00, 2.00)	1.00 (1.00, 2.00)	1.00 (1.00, 2.00)	0.089
Assisted conception (yes, %)	87 (5.06%)	62 (4.84%)	21 (5.97%)	4 (4.49%)	0.667
History of ICP (yes, %)	102 (5.93%)	65 (5.08%)	30 (8.52%)	7 (7.87%)	0.039
History of stillbirth (yes, %)	18 (1.05%)	9 (0.70%)	9 (2.56%)	0 (0)	0.015
Gestational week of onset of ICP (weeks)	36.10 (32.40, 38.40)	36.40 (33.00, 38.50)	35.40 (32.00, 38.10)	34.40 (30.30, 37.00)	<0.001
Pruritus (yes, %)	553 (32.13%)	266 (20.78%)	245 (69.60%)	42 (47.19%)	<0.001
TBA (µmol/L)	22.80 (14.20, 40.60)	17.85 (12.70, 25.20)	56.65 (46.00, 69.35)	130.70 (112.30, 159.00)	<0.001
AST (IU/L)	27.00 (18.00, 71.00)	25.00 (17.00, 55.00)	33.00 (19.00, 123.50)	94.00 (42.00, 239.50)	<0.001
ALT (IU/L)	19.00 (9.00, 92.00)	16.00 (9.00, 67.75)	28.00 (11.00, 166.00)	116.00 (38.50, 261.50)	<0.001
TBIL (µmol/L)	11.80 (9.20, 15.80)	11.40 (9.00, 14.80)	12.70 (9.80, 18.20)	19.40 (12.45, 27.25)	<0.001
DBIL (µmol/L)	4.50 (3.30, 6.60)	4.30 (3.30, 5.80)	5.00 (3.50, 9.40)	11.20 (5.05, 18.15)	<0.001
IBIL (µmol/L)	7.10 (5.30, 9.10)	7.10 (5.30, 9.10)	7.10 (5.40, 9.10)	7.00 (5.35, 9.25)	0.927
HDP (yes, %)	184 (10.69%)	137 (10.70%)	37 (10.51%)	10 (11.24%)	0.980
GDM (yes, %)	307 (17.84%)	237 (18.52%)	48 (13.64%)	22 (24.72%)	0.023
Thyroid dysfunction (yes, %)	76 (4.42%)	55 (4.30%)	18 (5.11%)	3 (3.37%)	0.710
PROM (yes, %)	284 (16.50%)	230 (17.97%)	43 (12.22%)	11 (12.36%)	0.020
Virus hepatitis (yes, %)	116 (6.74%)	84 (6.56%)	23 (6.53%)	9 (10.11%)	0.428

BMI: Body mass index; TBA: Total bile acids; AST: Aspartate transaminase; ALT: Alanine transaminase; TBIL: Total bilirubin; DBIL: Direct bilirubin; ICP: Intrahepatic cholestasis of pregnancy; IBIL: Indirect bilirubin; HDP: Hypertensive disorder of pregnancy; GDM: Gestational diabetes mellitus; PROM: Premature rupture of membranes.

### Study on the relationship between the severity of ICP and perinatal outcomes

Next, we conducted a detailed analysis of the relationship between the severity of ICP in pregnant women and the outcomes of their infants (Table 3). In neonatology, infants born with a birth weight below 2500 g (2.5 kg) are classified as low birth weight infants unless they are full-term but small for gestational age (SGA) infants [23]. The diagnosis of neonatal asphyxia is made when newborns exhibit symptoms and signs, such as respiratory distress, pallor or cyanosis, low muscle tone, decreased heart rate, and altered level of consciousness [24]. Findings revealed a significant decrease in gestational weeks (*P* < 0.001) and reduced birth weight in newborns (*P* < 0.001) with increasing severity of ICP, directly indicating the negative impact of ICP severity on gestational length and fetal growth. Concerning delivery methods, the rate of cesarean delivery significantly increased to 84.27% in severe ICP cases, compared to 64.69% in mild ICP cases, indicating a rising demand for cesarean delivery with increased ICP severity (*P* < 0.001). Additionally, the rate of meconium staining in amniotic fluid was highest in severe ICP patients at 29.21%, significantly higher than the 15.86% in the mild group, highlighting the increased fetal risks with escalating ICP severity (*P* < 0.001) (Table 3). Furthermore, the stillbirth rate and neonatal asphyxia rate were significantly higher in the severe ICP group compared to the mild and moderate groups, further confirming the significant correlation between ICP severity and adverse perinatal outcomes. Although there was no significant difference in iatrogenic preterm birth rates among different ICP severities (*P* ═ 0.298), the rate of spontaneous preterm birth significantly increased to 16.85% in severe ICP cases, underscoring the greater risk of natural preterm birth as ICP severity increases (Table 3).

**Table 3:** Outcomes of neonates born to mothers with ICP categorized by disease severity

	**ICP**	**Mild ICP**	**Moderate ICP**	**Severe ICP**	
**Outcomes**	***N* ═ 1721**	***N* ═ 1280**	***N* ═ 352**	***N* ═ 89**	***P* value**
Gestational age at delivery of ICP (weeks)	38.10 (37.00, 39.10)	38.30 (37.20, 39.20)	37.50 (36.10, 38.60)	36.30 (34.50, 37.55)	<0.001
Birth weight (kg)	3.08 (2.75, 3.40)	3.10 (2.80, 3.40)	2.99 (2.60, 3.30)	2.75 (2.32, 3.10)	<0.001
*Delivery mode*					
Vaginal delivery (yes, %)	528 (30.68%)	452 (35.31%)	62 (17.61%)	14 (15.73%)	<0.001
Cesarean delivery (yes, %)	1193 (69.32%)	828 (64.69%)	290 (82.39%)	75 (84.27%)	
*PTB*					
Iatrogenic PTB (yes, %)	307 (17.84%)	175 (13.67%)	98 (27.84%)	34 (38.20%)	<0.001
Spontaneous PTB (yes, %)	89 (5.17%)	50 (3.91%)	24 (6.82%)	15 (16.85%)	
MSAF (yes, %)	321 (18.65%)	203 (15.86%)	92 (26.14%)	26 (29.21%)	<0.001
Stillbirth (yes, %))	8 (0.46%)	4 (0.31%)	2 (0.57%)	2 (2.25%)	0.049
Neonatal asphyxia (yes, %)	30 (1.74%)	17 (1.33%)	8 (2.27%)	5 (5.62%)	0.011

PTB: Premature birth; MSAF: Meconium-stained amniotic fluid; ICP: Intrahepatic cholestasis of pregnancy.

**Table 4:** Risk assessment of perinatal outcomes in pregnancies affected by different grades of ICP

**Outcomes**	**Raw model**		**Adjusted model 1^a^**		**Adjusted model 2^b^**	
	**OR (95% CI)**	***P* value**	**OR (95% CI)**	***P* value**	**OR (95% CI)**	***P* value**
Mild ICP	Reference		Reference		Reference	
*PTB*						
Moderate ICP	2.49 (1.91, 3.23)	<0.001	2.50 (1.89, 3.31)	<0.001	2.51 (1.89, 3.33)	<0.001
Severe ICP	5.74 (3.69, 8.93)	<0.001	5.18 (3.23, 8.32)	<0.001	5.13 (3.19, 8.27)	<0.001
*P*-for-trend	<0.001		<0.001		<0.001	
Cesarean delivery						
Moderate ICP	2.55 (1.90, 3.44)	<0.001	2.75 (2.00, 3.78)	<0.001	2.74 (2.00, 3.76)	<0.001
Severe ICP	2.92 (1.63, 5.23)	<0.001	3.68 (1.91, 7.10)	<0.001	3.60 (1.87, 6.95)	<0.001
*P*-for-trend	<0.001		<0.001		<0.001	
*FGR*						
Moderate ICP	1.06 (0.60, 1.87)	0.84	0.90 (0.48, 1.67)	0.74	0.79 (0.41, 1.53)	0.49
Severe ICP	1.90 (0.84, 4.31)	0.12	2.01 (0.88, 4.63)	0.10	1.70 (0.69, 4.20)	0.25
*P*-for-trend	0.31		0.22		0.35	
Neonatal asphyxia						
Moderate ICP	1.73 (0.74, 4.05)	0.20	1.87 (0.75, 4.64)	0.18	1.85 (0.74, 4.63)	0.19
Severe ICP	4.52 (1.63, 12.55)	<0.01	5.81 (2.00, 16.88)	0.001	5.47 (1.87, 16.02)	0.002
*P*-for-trend	0.01		0.01		0.01	
**Outcomes**	**Adjusted Model 3^c^**		**Adjusted Model 4^d^**		**Adjusted Mode 5^e^**	
	**OR (95% CI)**	***P* value**	**OR (95% CI)**	***P* value**	**OR (95% CI)**	***P* value**
Mild ICP	Reference		Reference		Reference	
*PTB*						
Moderate ICP	2.51 (1.90, 3.32)	<0.001	2.51 (1.89, 3.32)	<0.001	2.51 (1.89 3.32)	<0.001
Severe ICP	5.16 (3.22, 8.29)	<0.001	5.21 (3.25, 8.37)	<0.001	5.15 (3.21, 8.26)	<0.001
*P*-for-trend	<0.001		<0.001		<0.001	
*Cesarean delivery*						
Moderate ICP	2.76 (2.01, 3.80)	<0.001	2.75 (2.00, 3.78)	<0.001	2.75 (2.01, 3.78)	<0.001
Severe ICP	3.66 (1.90, 7.06)	<0.001	3.70 (1.92, 7.13)	<0.001	3.70 (1.92, 7.14)	<0.001
*P*-for-trend	<0.001		<0.001		<0.001	
*FGR*						
Moderate ICP	0.90 (0.48, 1.67)	0.74	0.90 (0.48, 1.68)	0.75	0.90 (0.48, 1.67)	0.74
Severe ICP	2.01 (0.87, 4.64)	0.10	2.10 (0.91, 4.85)	0.08	2.03 (0.88, 4.70)	0.10
*P*-for-trend	0.22		0.18		0.21	
*Neonatal asphyxia*						
Moderate ICP	1.81 (0.73, 4.51)	0.20	1.87 (0.75, 4.66)	0.18	1.88 (0.75, 4.67)	0.18
Severe ICP	6.41 (2.19, 18.74)	0.001	5.98 (2.05, 17.44)	0.001	5.81 (2.00, 16.89)	0.001
*P*-for-trend	0.003		<0.01		<0.01	

^a^Adjusted for mother's age, number of pregnancies, number of deliveries, previous history of ICP, BMI, and mode of conception. ^b^Adjusted Model 1 + mother HDP. ^c^Adjusted Model 1 + mother GDM. ^d^Adjusted Model 1 + thyroid disease in mothers. ^e^Adjusted Model 1 + viral hepatitis in mothers. PTB: Meconium-stained amniotic fluid; MSAF: Meconium-stained amniotic fluid; ICP: Intrahepatic cholestasis of pregnancy, FGR: Fetal growth restriction.

### Analysis of the relationship between the severity of ICP and the risk of adverse perinatal outcomes

In this study, we investigated the relationship between different levels of ICP and adverse neonatal outcomes using logistic regression analysis, with mothers with mild ICP serving as the control group. In the raw model, when mothers had moderate ICP, the risk of preterm birth and cesarean delivery in neonates was 2.49 times [95% CI: (1.91, 3.23)] and 2.55 times [95% CI: (1.90, 3.44)] higher, respectively, compared to the control group. When mothers had severe ICP, the risks of preterm birth and cesarean delivery in neonates increased to 5.74 times [95% CI: (3.69, 8.93)] and 2.92 times [95% CI: (1.63, 5.23)] respectively (Table 4). Furthermore, with each increment in ICP severity level, both risks exhibited a rising trend (*P*-for-trend <0.0001). Model 1, adjusted for maternal age, gravidity, parity, history of ICP, BMI, and mode of conception, minimally influenced the results. In the adjusted Model 1, the association between moderate ICP in mothers and neonatal asphyxia was not statistically significant, whereas mothers with severe ICP had a 5.81 times higher risk of neonatal asphyxia compared to the control group [95% CI: (2.00, 16.88)] (Table 4). After further adjustment for HDP, gestational diabetes, thyroid disease, and viral hepatitis in Model 1, all the aforementioned associations remained significant. However, there was no statistical association between maternal moderate or severe ICP and FGR (Table 4).

In various adjustment models, the significant impact of severe ICP on the risk of cesarean delivery is particularly pronounced, with odds ratios ranging between 3.60 and 3.70, further underscoring the stability of this association (Table 4). However, unlike preterm birth and cesarean delivery, our analysis of FGR did not reveal a significant association with moderate ICP. Although extreme ICP appears to slightly elevate the risk of FGR, the correlation did not reach statistical significance in any of the models, suggesting a relatively weaker influence of ICP severity on FGR (Table 4). Regarding neonatal asphyxia, we observed a tendency for severe ICP to increase the risk of neonatal asphyxia compared to mild ICP, but the correlation was not statistically significant. Nonetheless, extreme ICP significantly raised the risk of neonatal asphyxia, with consistent and significant odds ratios across different models, further strengthening the reliability of this association (Table 4).

### Literature screening process and quality assessment of included studies

**Figure 2.**
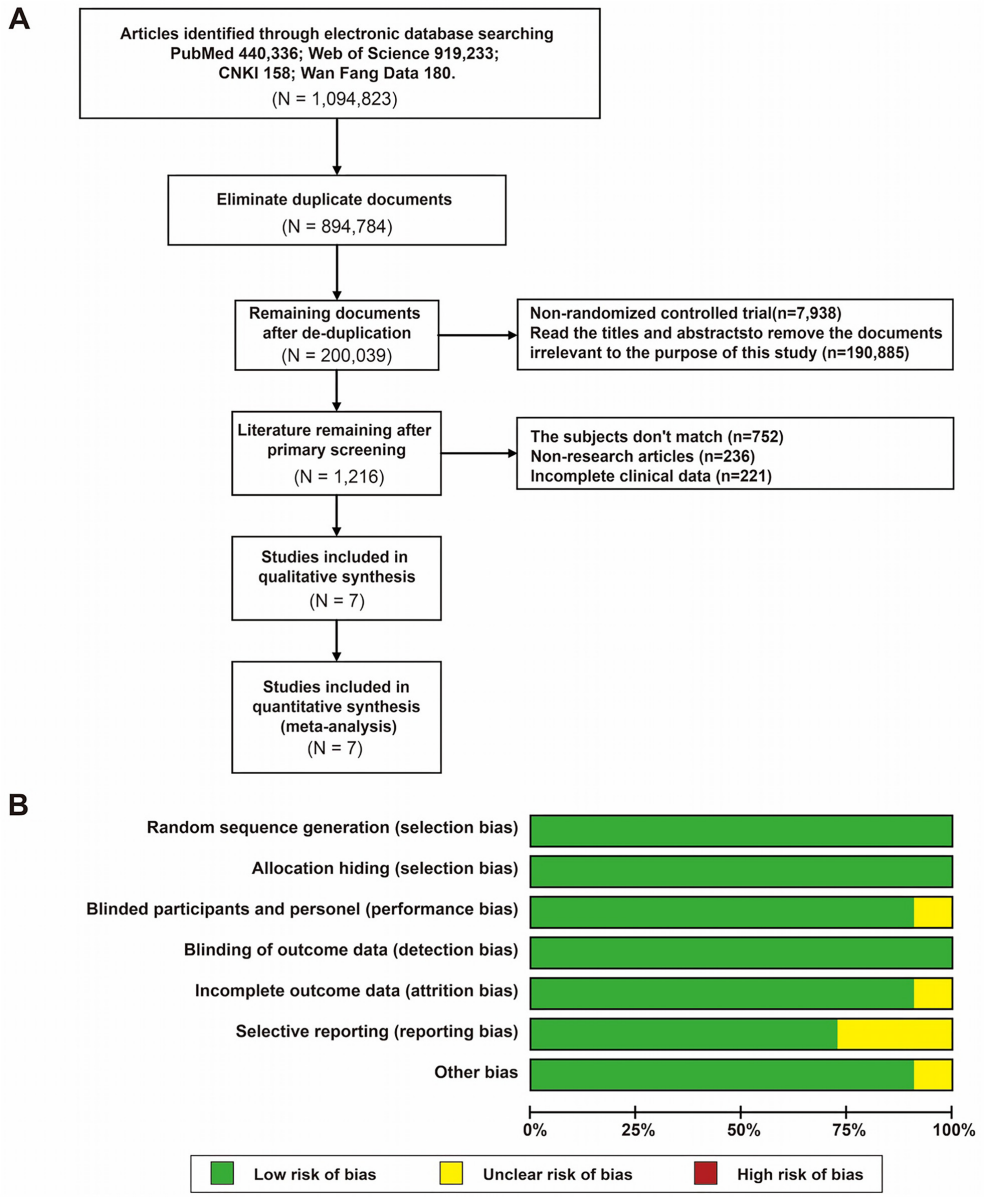
**Literature screening process and summary of quality assessment results for included studies.** (A) Flowchart of literature inclusion; (B) Summary of bias risk assessment for included studies.

In this systematic review and meta-analysis, rigorous literature screening criteria were implemented to ensure the quality and accuracy of the studies (Figure 2A). Additionally, this study evaluated the quality of the included literature based on the Cochrane risk of bias tool standards (Figure 2B). Potential biases within each study, such as random sequence generation, allocation concealment, blinding, and outcome assessment, were categorized into levels of low, high, or unclear risks. Studies were classified as having a high risk of selection bias in cases of inadequate randomization or allocation concealment measures. Instances of missing information or insufficient data to assess bias risk were considered unclear, while studies with proper randomization, allocation concealment, and blinding procedures were categorized as low bias risk. Through this systematic assessment process, our study aimed to ensure that each included study underwent a comprehensive and fair quality appraisal.

### Association between biochemical marker abnormalities in ICP patients and adverse pregnancy outcomes

Our meta-analysis focused on three critical biochemical markers in ICP: TBA, transaminases, and bilirubin. These markers are closely associated with pregnancy outcomes in ICP patients. We detailed the level changes of these markers and discussed their potential impact on pregnancy outcomes.

**Figure 3.**
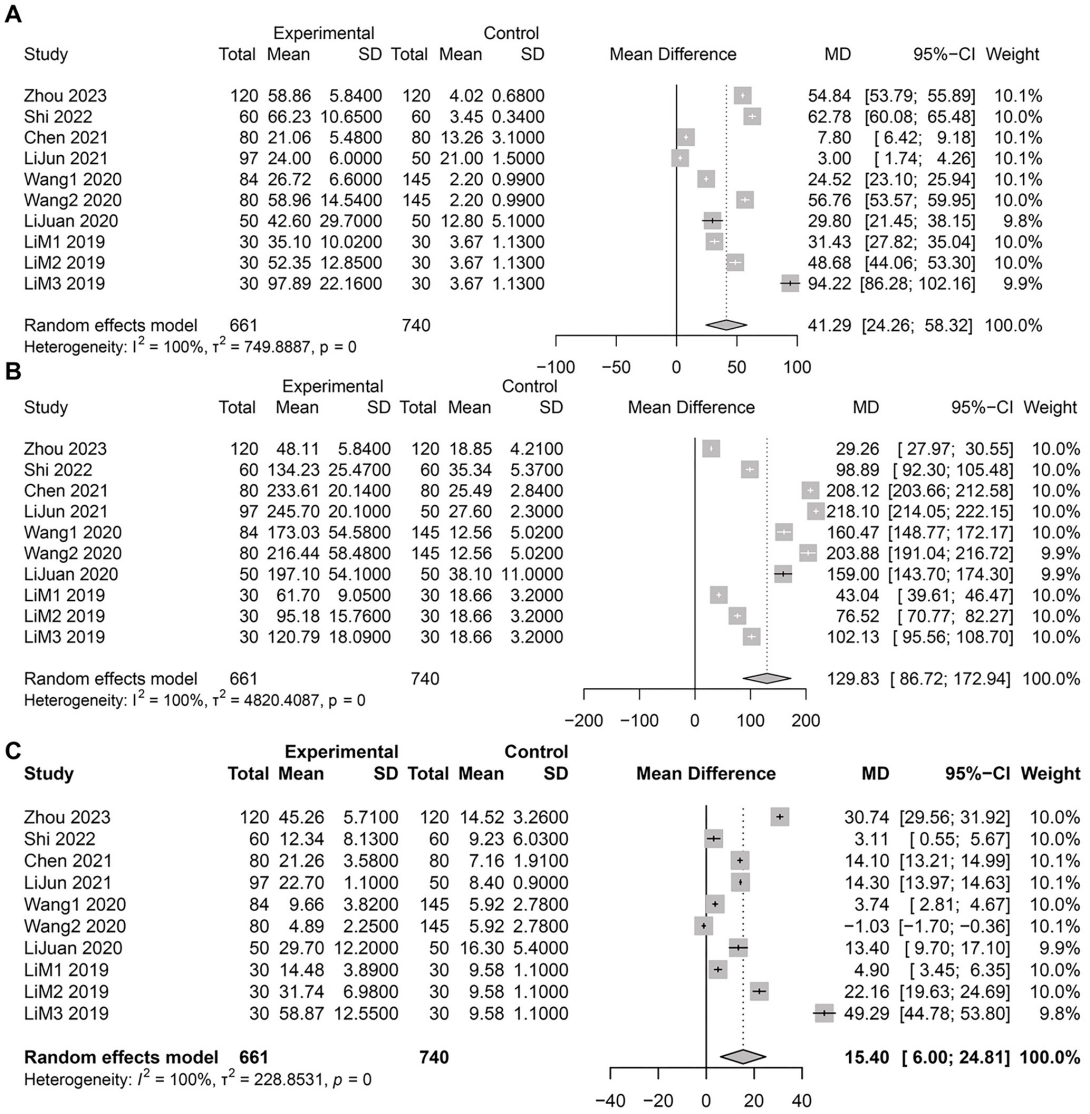
**Meta-analysis results on the correlation between biochemical marker levels in ICP patients and pregnancy outcomes.** (A) Forest plot comparing TBA levels between ICP patients and healthy pregnant women; (B) Forest plot of transaminase level differences in ICP patients; (C) Forest plot of bilirubin level differences in ICP patients. ICP: Intrahepatic cholestasis of pregnancy; TBA: Total bile acids.

Analysis of TBA levels in ICP patients revealed a significant difference compared to healthy pregnant women (Figure 3A). Pooled data indicated that the average TBA level in the ICP group was notably higher than in the healthy pregnancy group, with a mean difference (MD) of 41.29 µmol/L and a 95% CI of [24.26; 58.32]. Similarly, results for transaminase levels showed significant elevations in ICP patients compared to the control group (Figure 3B), suggesting more pronounced liver dysfunction in ICP patients. The pooled MD was 129.83, with a 95% CI of [86.72; 172.94]. Regarding bilirubin levels, data aggregation showed that ICP patients also had significantly higher bilirubin levels than healthy pregnant women (Figure 3C). The combined MD was 15.40, with a 95% CI of [6.00; 24.81].

### Robust association between biochemical markers in ICP patients and pregnancy outcomes

**Figure 4.**
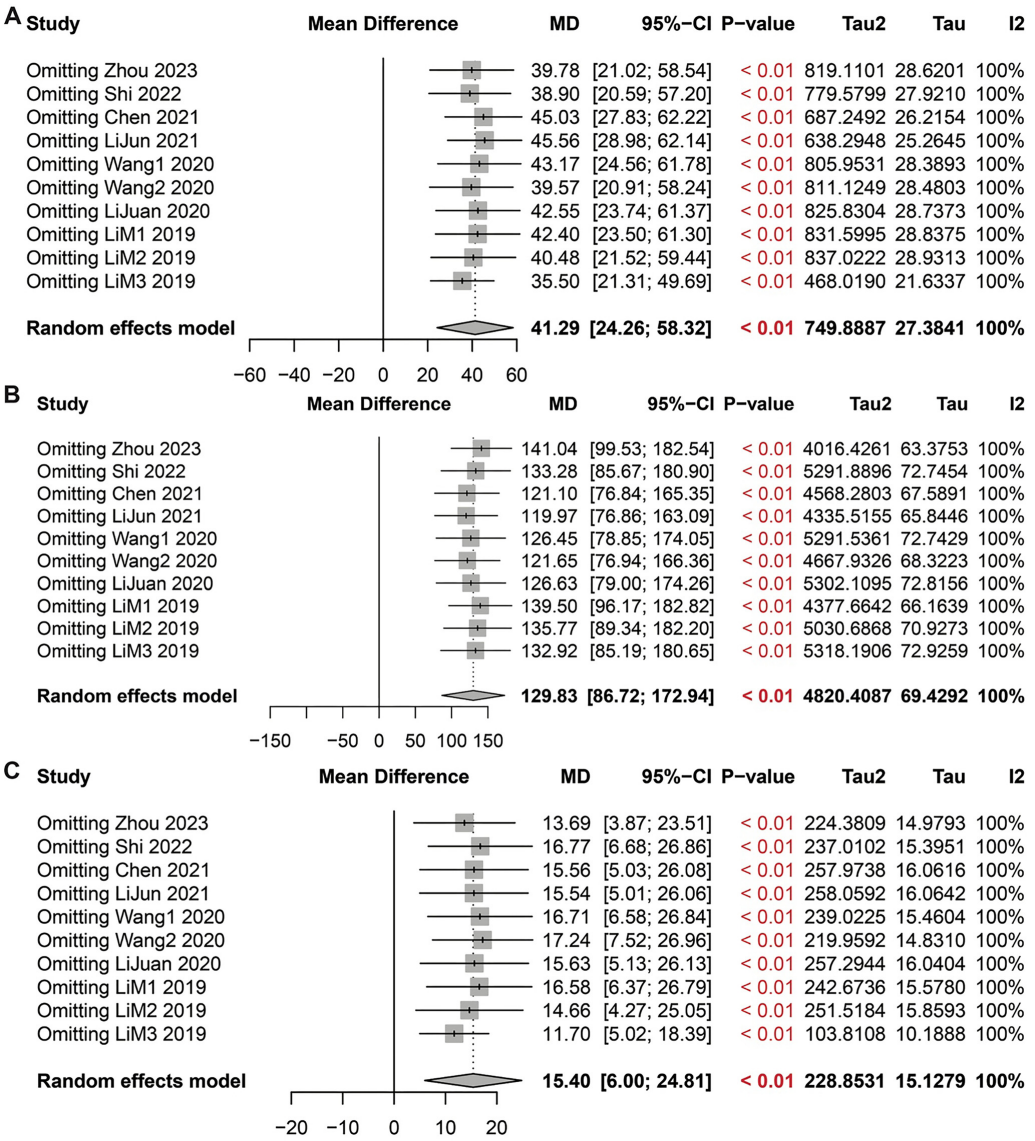
**Sensitivity analysis forest plots.** (A) Forest plot for sensitivity analysis of TBA levels in ICP patients; (B) Forest plot for sensitivity analysis of transaminase levels in ICP patients; (C) Forest plot for sensitivity analysis of bilirubin levels in ICP patients. ICP: Intrahepatic cholestasis of pregnancy; TBA: Total bile acids.

In our meta-analysis of TBA, transaminases, and bilirubin levels in patients with ICP, sensitivity analyses were conducted to assess the impact of excluding each study on the overall effect size (Figure 4). This step allowed us to evaluate whether the pooled MD and 95% CIs underwent significant changes when specific studies were excluded from the total analysis.

For TBA levels, it was observed that the pooled effect size and 95% CIs remained significant regardless of which study was excluded, indicating that TBA levels in ICP patients are generally higher than in healthy pregnant women and are associated with adverse pregnancy outcomes (Table 5 and Figure 4A). Sensitivity analyses of transaminase levels revealed a similar trend. The changes in the pooled effect size and CIs were minimal after excluding any single study, supporting the observation that transaminase levels are elevated in ICP patients (Table 6 and Figure 4B). Likewise, sensitivity analyses for bilirubin levels demonstrated that the difference in bilirubin levels between ICP patients and healthy pregnant women remained significant even after excluding any one study, underscoring the stable and vital role of bilirubin in the pregnancy outcomes of ICP (Table 7 and Figure 4C).

**Table 5:** Sensitivity analysis of TBA level in ICP patients

**Deleted document**	**Pooled effect size of remaining literature (Pooled MD)**	**95% confidence intervals (CI) for the remaining literature**
Zhou, 2023	39.7836	[21.0234; 58.5437]
Shi, 2022	38.8958	[20.5929; 57.1986]
Chen, 2021	45.0284	[27.8339; 62.2228]
LiJun, 2021	45.5599	[28.9842; 62.1357]
Wang1, 2020	43.1717	[24.5621; 61.7813]
Wang2, 2020	39.5734	[20.9073; 58.2395]
LiJuan, 2020	42.5538	[23.7396; 61.3680]
LiM1, 2019	42.3978	[23.5000; 61.2956]
LiM2, 2019	40.4801	[21.5239; 59.4364]
LiM3, 2019	35.5030	[21.3143; 49.6918]

ICP: Intrahepatic cholestasis of pregnancy; TBA: Total bile acids.

In summary, despite the high heterogeneity among studies, sensitivity analyses confirmed the high stability of our meta-analysis results. Although individual studies may impact the magnitude of the results, the overall trend remains unchanged, providing solid scientific support for the clinical management of ICP patients.

### Publication bias assessment of biochemical markers in ICP patients

**Figure 5.**
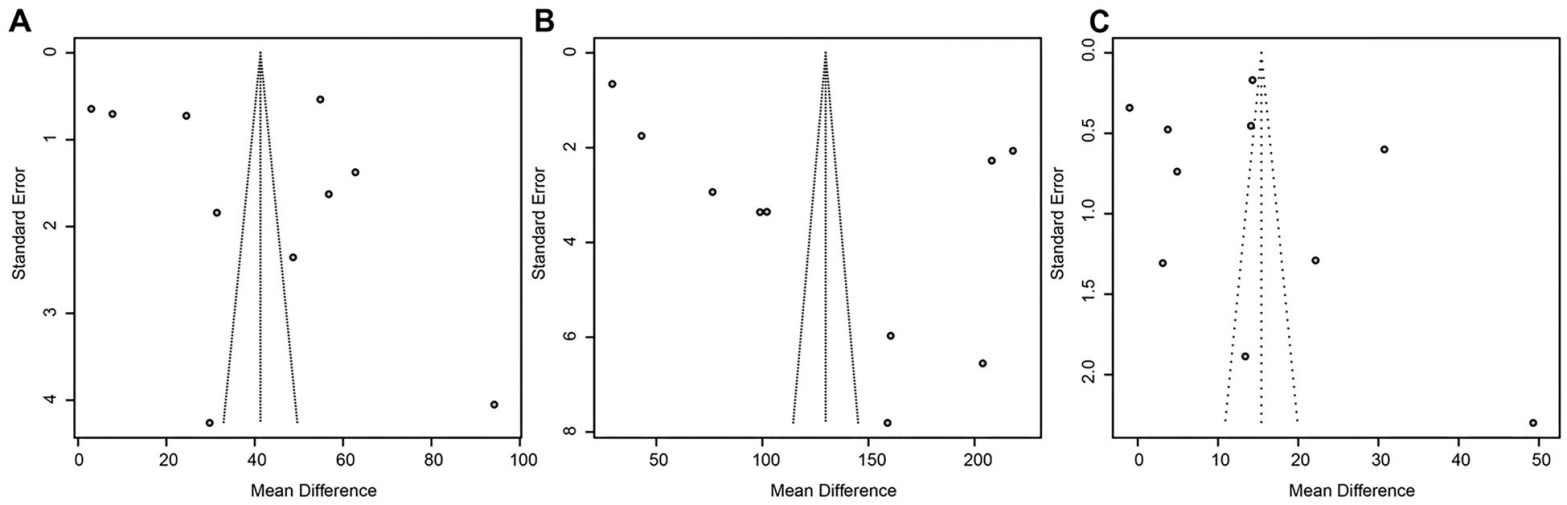
**Funnel plots for publication bias assessment.** (A) Funnel plot for publication bias in the meta-analysis of TBA levels in ICP patients; (B) Funnel plot for publication bias in the meta-analysis of transaminase levels in ICP patients; (C) Funnel plot for publication bias in the meta-analysis of bilirubin levels in ICP patients. ICP: Intrahepatic cholestasis of pregnancy; TBA: Total bile acids.

In our meta-analysis investigating the relationship between biochemical markers in ICP patients and pregnancy outcomes, assessing publication bias was crucial. We assessed whether unpublished studies influenced the overall data distribution by analyzing funnel plots, which examine the relationship between the MD and standard error. This visual inspection revealed the symmetry of data point distribution, aiding in evaluating potential publication bias (Figure 5).

**Table 6:** Sensitivity analysis of transaminase level in ICP patients

**Deleted document**	**Pooled effect size of remaining literature (Pooled MD)**	**95% CI for the remaining literature**
Zhou, 2023	13.6901	[3.8717; 23.5084]
Shi, 2022	16.7702	[6.6843; 26.8562]
Chen, 2021	15.5578	[5.0342; 26.0814]
LiJun, 2021	15.5356	[5.0099; 26.0613]
Wang1, 2020	16.7099	[6.5779; 26.8418]
Wang2, 2020	17.2384	[7.5161; 26.9608]
LiJuan, 2020	15.6326	[5.1303; 26.1349]
LiM1, 2019	16.5796	[6.3718; 26.7873]
LiM2, 2019	14.6609	[4.2724; 25.0494]
LiM3, 2019	11.7037	[5.0177; 18.3898]

ICP: Intrahepatic cholestasis of pregnancy; CI: Confidence intervals.

**Table 7:** Sensitivity analysis of bilirubin levels in ICP patients

**Deleted document**	**Pooled effect size of remaining literature (Pooled MD)**	**95% confidence intervals (CI) for the remaining literature**
Zhou, 2023	13.6901	[3.8717; 23.5084]
Shi, 2022	16.7702	[6.6843; 26.8562]
Chen, 2021	15.5578	[5.0342; 26.0814]
LiJun, 2021	15.5356	[5.0099; 26.0613]
Wang1, 2020	16.7099	[6.5779; 26.8418]
Wang2, 2020	17.2384	[7.5161; 26.9608]
LiJuan, 2020	15.6326	[5.1303; 26.1349]
LiM1, 2019	16.5796	[6.3718; 26.7873]
LiM2, 2019	14.6609	[4.2724; 25.0494]
LiM3, 2019	11.7037	[5.0177; 18.3898]

ICP: Intrahepatic cholestasis of pregnancy; CI: Confidence intervals.

In analyzing TBA levels, although some data points diverged from the central axis, most studies aligned within the expected symmetrical distribution (Figure 5A). This pattern indicates that despite a few outliers, the overall increase in TBA levels among ICP patients and its association with adverse pregnancy outcomes is not significantly impacted by publication bias, enhancing the credibility of the findings for clinical reference. A similar funnel plot analysis for the relationship between transaminases and ICP (Figure 5B) revealed asymmetrical data point distribution, suggesting potential publication bias. However, the consistency in effect sizes across both large and small sample studies suggests a stable phenomenon of elevated transaminase levels in ICP patients significantly affecting pregnancy outcomes, warranting caution towards potential publication bias. The bilirubin level analysis through funnel plots indicated some asymmetry, suggesting a risk of publication bias (Figure 5C). Yet, the presence of studies with larger effect sizes and standard errors does not sufficiently challenge the overall correlation between elevated bilirubin levels and adverse pregnancy outcomes. Future research should include more unpublished or smaller scale studies to ensure the comprehensiveness and accuracy of the analysis.

## Discussion

ICP, the most common liver disease during pregnancy, typically presents with skin itching and elevated bile acid and alanine aminotransferase levels in the third trimester [25–27]. This condition not only causes discomfort to pregnant women but also significantly increases the risk of perinatal complications, such as preterm birth, respiratory distress, and stillbirth [18, 28, 29]. The etiology of ICP is complex, involving changes in pregnancy hormone levels, genetic factors, and abnormalities in hepatobiliary transport proteins, with mutations in MDR3 and BSEP playing a significant role [30]. Recent studies on the pathophysiological mechanisms of ICP have highlighted the involvement of various factors, especially the critical role of the estrogen-bile acid axis in the disease’s progression [25, 31, 32]. Additionally, variations in the gut microbiome have been found to significantly affect ICP development by inhibiting FXR signaling and altering bile acid metabolism, thus triggering the onset of the disease [33, 34]. The primary treatment for ICP involves using ursodeoxycholic acid (UDCA) to lower bile acid levels and alleviate itching [26, 28, 35]. However, compared to placebo, UDCA has a minimal effect on improving itch scores, suggesting the need for further evaluation and development of more effective treatment strategies [36]. The overall incidence rate of ICP diagnosed as singleton is approximately 1.28%. Mild ICP patients exhibit normal or slightly elevated levels of BAs, and milder symptoms, such as mild itching and less severe bile accumulation. In moderate cases, BA levels increase, and symptoms intensify gradually with noticeable itching and progressive bile backup but without reaching severe levels. Severe cases show significantly elevated BA levels, severe clinical symptoms like intense and persistent itching, possible accompanying complications, and severe impact of bile accumulation on the health of the pregnant woman and the fetus [37]. ICP is also associated with abnormalities in maternal lipid metabolism, indicating that pregnant women with ICP have a higher risk of lipid dysregulation, which underscores the importance of a comprehensive understanding of ICP’s pathological mechanisms [38, 39]. Moreover, metabolomics has provided new insights into ICP research, revealing fundamental changes in bile acid and lipid metabolism processes [40].

The results of the meta-analysis in this study indicate that levels of TBA, transaminases, and bilirubin in patients with ICP are significantly higher than in healthy pregnant women. The high degree of heterogeneity suggests that while the conclusions are generalizable, there may be differences in diagnosis, measurement, and management among different studies. Therefore, in clinical practice, special attention should be paid to these biochemical markers in ICP patients to identify and manage potential risks during pregnancy. Furthermore, the elevation of TBA, bilirubin, and transaminase levels in ICP patients is significantly correlated with adverse pregnancy outcomes. Despite some degree of data point deviation observed in the publication bias analysis for each indicator, the overall conclusions remain robust. These findings underscore the importance of monitoring and controlling these biochemical markers in the management of ICP patients. To further ensure the accuracy of the conclusions, future studies should consider including more unpublished or small-scale studies to enhance our understanding of the relationship between these markers and the outcomes of ICP pregnancies.

In this retrospective case analysis, we present the clinical features and perinatal outcomes of singleton pregnancies with ICP in the Jiangxi region of China. The incidence of ICP in singleton pregnancies in this study was 1.28%. Over the past six years, the incidence of ICP in Jiangxi has shown an increasing trend, particularly notable for mild bile acid accumulation. With advancing ICP severity, patients experienced earlier onset of symptoms, elevated peak serum levels of AST, ALT, TBIL, and DBIL, and increased itching. More importantly, we observed that the severity of ICP was closely associated with gestational age at delivery, mode of delivery, MSAF, and birth weight. As ICP severity increased, the risk of adverse outcomes, such as preterm birth and cesarean delivery significantly escalated, while the risk of neonatal asphyxia notably increased only when TBA levels reached 100 µmol/L.

sTBA is the earliest abnormal biochemical marker in ICP and is closely linked to adverse perinatal outcomes [26, 41, 42]. There is a consensus that fasting sTBA ≥10 µmol/L [20] or random sTBA ≥19 µmol/L are diagnostic criteria for ICP [6, 43], with sTBA peak values of 40 and 100 µmol/L serving as gradation thresholds for ICP severity. Based on the diagnostic criterion of fasting sTBA ≥10 µmol/L and the mentioned thresholds [41, 42], this study graded 1721 ICP patients, finding that serum levels of AST, ALT, TBIL, and DBIL significantly increased with ICP severity. These findings underscore a close correlation between serum sTBA and these biochemical markers, suggesting that in diagnosing and assessing ICP severity, it is necessary to consider transaminase and bilirubin levels comprehensively for a more thorough evaluation of the patient’s condition [44].

According to the 2015 guidelines of the Chinese Medical Association, the gestational age at onset is a crucial indicator for assessing the severity of ICP [20]. Multiple studies have established a close relationship between the onset time of ICP, sTBA levels, and adverse perinatal outcomes. For instance, research by Zhou et al. [45] found that ICP patients with onset before 28 weeks of pregnancy had higher sTBA levels (41 vs. 32 µmol/L), higher rates of preterm birth (33.3% vs. 15.6%), cesarean delivery (91.7% vs. 78.6%), and neonatal asphyxia (14.6% vs. 5.4%). Uyar et al.’s [46] study noted lower birth weights in newborns from ICP patients with onset after 32 weeks (2924.3 vs. 3135.6 g, *P* ═ 0.035). Estiú’s research also indicated a higher risk of MSAF with early-onset ICP [47]. Research by Oztekin et al. [48] confirmed that TBA levels (OR 1.04; 95% CI 1.01–1.08) and exposure duration (OR 1.11; 95% CI 1.05–1.17) are crucial predictors of fetal asphyxia in ICP. Consistent with previous findings, our study revealed that earlier-onset ICP patients exhibited higher sTBA peaks associated with more adverse perinatal outcomes despite immediate treatment with UDCA upon diagnosis. Thus, the timing of ICP onset holds significant value in predicting disease progression and assessing condition severity, emphasizing the importance of obstetricians considering the onset timing of ICP when evaluating patient history.

ICP poses a severe risk to perinatal health, with primary dangers, including preterm birth, fetal distress, MSAF, and stillbirth [18, 28, 49]. Previous research consistently shows that higher ICP grades correlate with increased rates of preterm birth. For example, a population-based prospective case-control study confirmed that severe ICP significantly elevates the risk of preterm birth compared to non-ICP cases (25% vs. 6.5%; adjusted OR 5.39, 95% CI 4.17–6.98) [50]. Moreover, a meta-analysis revealed escalating preterm birth rates with increasing ICP severity: <40 µmol/L, 23.25%; 40–99 µmol/L, 28%; and ≥100 µmol/L, 47%, with a higher odds ratio for preterm birth in ICP women (OR 3.54 [95% CI 2.72–4.62]) [15]. Consistent with previous research, our study also indicates that the moderate ICP group and the severe ICP group have a higher preterm birth rate compared to the mild ICP group. Additionally, with the increasing severity of ICP, the probability of preterm birth in the perinatal period also increases. One possible explanation is that both healthcare providers and patients are overly concerned about the potential harm of elevated sTBA levels to the perinatal outcomes and thus opt for earlier interventions. Our study reveals a cesarean delivery rate as high as 69.32% among ICP perinates, particularly with the moderate and severe ICP groups surpassing 80% in cesarean rates, significantly higher than those reported by Chen et al. [11] and Kong et al. [51] for ICP cesarean rates. While cesarean delivery is relatively safe for high-risk pregnant women and high-risk perinates, excessive use of cesarean delivery could endanger the short-term and long-term health of children [52–55]. Therefore, a more cautious approach will be necessary in future practice.

Neonatal asphyxia is a significant factor affecting the prognosis of perinates, potentially leading to various neonatal complications, and even mortality [56]. According to Xu et al. [57], the rates of neonatal asphyxia in the mild, moderate, and severe ICP groups were 1.0%, 2.0%, and 3.1%, respectively, lower than those reported in this study, possibly due to the more proactive medical interventions for ICP patients at the center of Xu’s study. Furthermore, Cui et al.’s analysis of neonatal asphyxia rates across different levels of ICP revealed an increased incidence in the moderate ICP group compared to the mild ICP group (pooled RR 1.67; 95% CI 1.18–2.36) [58, 59]. Our study also reached a similar conclusion; however, the findings indicated that the risk of neonatal asphyxia significantly increases only when sTBA levels reach 100 µmol/L and above. It is important to note that the assessment of neonatal asphyxia in this study was based solely on the Apgar score, which may not fully capture the severity of asphyxia. Future studies should incorporate cord gas analysis to provide a more comprehensive evaluation of neonatal asphyxia. This discovery provides crucial guidance for clinical diagnosis and treatment, contributing to the enhancement of perinatal outcomes.

**Figure 6.**
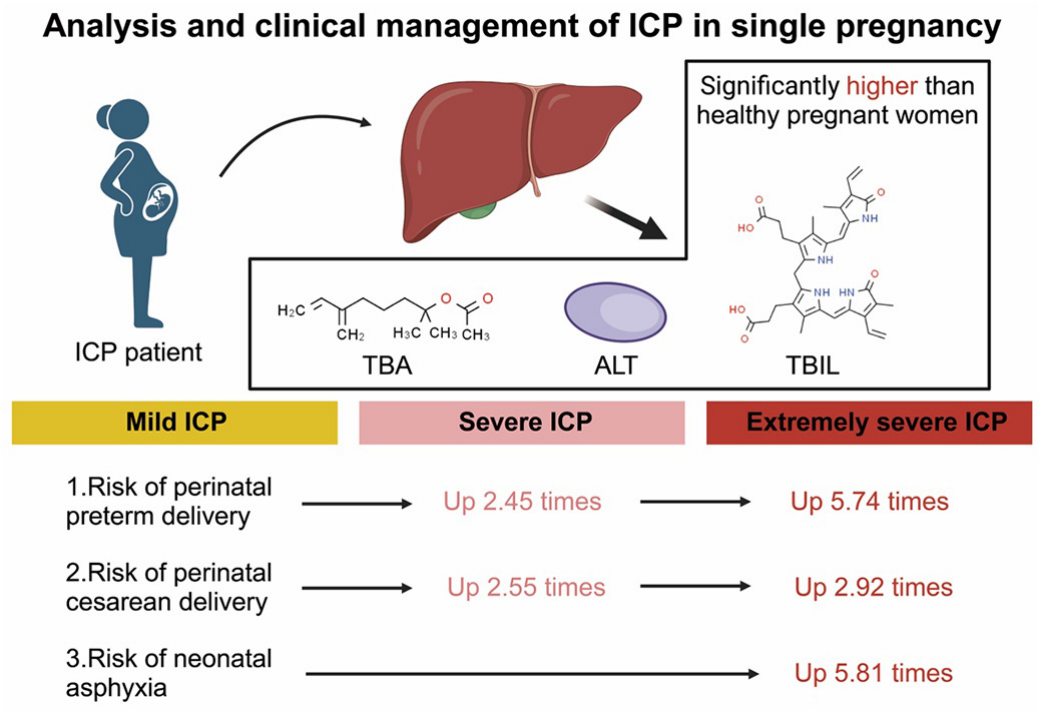
**Analysis of the impact of singleton pregnancy ICP and guidance for clinical management**. ICP: Intrahepatic cholestasis of pregnancy; TBA: Total bile acids; ALT: Alanine transaminase; TBIL: Total bilirubin.

It is crucial to note that while we discuss various key parameters independently in perinatal outcomes, such as premature delivery, stillbirth rates, and neonatal asphyxia rates, there often exist interrelations between different perinatal outcomes. For instance, premature infants, due to their incomplete development and especially poor lung function, are prone to respiratory distress and neonatal asphyxia issues. Therefore, there may be some correlation between these conditions.

The primary challenge of ICP for patients and obstetricians lies in the difficulty of completely avoiding the occurrence of sudden intrauterine fetal demise, even with meticulous fetal monitoring measures in place. A prospective case-control study involving 669 cases of severe ICP revealed a fetal demise rate of 1.5% in the severe ICP group. Furthermore, Ovadia’s research categorized ICP patients with TBA levels ≥40 µmol/L into moderate and severe groups, demonstrating fetal demise rates of 0.28% (95% CI 0.08–0.72) and 3.44% (95% CI 2.05–5.37), respectively. Compared to previous studies, our research indicates lower overall fetal demise rates in both moderate and severe ICP groups, particularly in the severe ICP group. This may be attributed to the timely hospitalization of these patients in our center, where UDCA combined with tauroursodeoxycholic acid treatment was administered, with most patients undergoing cesarean delivery after 36 weeks of gestation.

This study, conducted at the largest perinatal medical center in Jiangxi province, utilized its extensive delivery volume and typical high-risk pregnancy cases to retrospectively analyze clinical data of 1721 singleton pregnancies with ICP from October 2016 to December 2022 (Figure 6). The strengths of this study include a large sample size providing substantial data support for evaluating adverse outcomes in singleton pregnancy ICP infants, data sourced solely from electronic medical records ensuring information authenticity and result reliability, and the use of logistic regression and trend analysis considering maternal clinical characteristics and pregnancy complications to enhance the study’s depth and breadth. Despite offering valuable insights, the study has limitations. Its retrospective design may lead to underestimation of peak TBA values in moderate and severe ICP groups due to the potential pretreatment of study subjects before the consultation. Furthermore, limited resources for umbilical artery blood gas analysis prevented the comprehensive implementation of this test, prompting the use of Apgar scoring to assess neonatal asphyxia. The Apgar scores at 1-min primarily reflect the infant’s immediate condition after birth, while the 5-min Apgar scores are used to evaluate the newborn’s adaptation to the environment postnatally. Scoring’s subjectivity, influenced by the individual experience and competence of the raters, may introduce a level of subjectivity to the assessment. Additionally, while Apgar scoring rapidly evaluates neonatal health status post-birth, it may not fully reflect the infant’s condition throughout the entire delivery process. It is important to note that the severity of ICP represents a persistent functional impairment, with increasing severity resulting in more aberrant biochemical markers, such as TBA, transaminases, and bilirubin levels. Despite our rigorous severity classification criteria for ICP, ongoing variables could still impact experimental outcomes. Nevertheless, this study offers significant guidance for the clinical management of ICP, particularly in comprehensive assessment, proactive treatment, and scientific delivery planning, holding vital importance in enhancing perinatal outcomes.

Moreover, the results of the meta-analysis reveal that patients with ICP exhibit significantly higher levels of TBA, transaminases, and bilirubin compared to healthy pregnant women. The abnormalities in these biochemical markers are robustly correlated with adverse pregnancy outcomes. Sensitivity analysis and publication bias tests further validate the stability and reliability of these conclusions, offering a scientific basis for the more effective clinical management of ICP patients. Elevated levels and prolonged duration of TBA may increase the risk of neonatal asphyxia by enhancing bile acid toxicity, affecting lung development, and leading to conditions like amniotic fluid embolism [60, 61]. It is important to note the complexity and intimacy of the symbiotic relationship between the mother and the fetus, indicating continuous interdependency rather than mere independent coexistence. For example, during this study, the abnormal maternal state resulting from ICP significantly impacts the fetus through deviations in biochemical markers such as elevated levels of TBA, transaminases, and bilirubin. To better understand the effect of ICP as an independent variable on fetal pregnancy outcomes, this study examines it as a standalone event.

In conclusion, ICP poses risks not only to pregnant women but also can have serious health consequences for fetuses. Therefore, current research priorities are enhancing the accuracy of ICP diagnoses and developing more effective treatment methods. Future studies need to delve deeper into the pathophysiological mechanisms of ICP, especially the interaction between the gut microbiome and ICP, and how this knowledge can be translated into more effective treatment strategies. A comprehensive understanding of ICP can be achieved by delving into the relationship between gut microbiota and ICP. Studying the influence of gut microbiota on the development of ICP aids in grasping the pathogenesis of the disease and elucidating the roles played by the gut–liver axis and gut–bile axis in the occurrence and progression of ICP. Exploring the role of gut microbiota in ICP offers novel perspectives for prevention and intervention. Modulating the balance of gut microbiota or utilizing probiotics can impact the development and progression of ICP. Gut microbiota may serve as potential biomarkers or diagnostic indicators for ICP, potentially offering a new avenue for early disease detection [33, 62, 63]. Considering individual differences, personalized medicine may be vital to improving the management and prognosis of ICP. With ongoing research and the application of new technologies, we can anticipate a better understanding of ICP’s complexities and provide superior treatment options for patients.

## Conclusion

In conclusion, our extensive investigation into ICP underscores the pivotal role of biochemical markers in predicting and managing adverse pregnancy outcomes. Through a combination of retrospective case analysis and a meta-analysis spanning several years, we have identified a clear correlation between elevated levels of TBA, transaminases, and bilirubin in ICP patients and a higher risk of preterm birth, cesarean delivery, and neonatal asphyxia. Our findings highlight the increasing incidence of ICP, particularly mild cases, emphasizing the necessity for vigilant monitoring and early intervention.

This study not only reiterates the importance of a comprehensive approach to the clinical management of ICP but also calls for a broader collection and analysis of data to refine prevention, diagnosis, and treatment strategies. By focusing on the intricate relationship between biochemical marker abnormalities and ICP severity, we advocate for personalized treatment plans tailored to the individual clinical profiles of pregnant women, aiming to minimize the risk of adverse perinatal outcomes.

Furthermore, the study opens avenues for future research into the pathophysiological mechanisms of ICP, particularly the interactions between the gut microbiome and ICP, and the potential for developing more effective treatment modalities. Emphasizing the need for ongoing research and the application of new technologies, we envision a future where a deeper understanding of ICP’s complexities can lead to improved health outcomes for both pregnant women and their newborns.

As we continue to unravel the intricacies of ICP and its impact on pregnancy outcomes, our work lays a foundational stone for enhancing clinical practices and fostering a safer pregnancy journey for affected individuals. It is our hope that the insights garnered from this research will contribute significantly to the field of obstetrics, guiding clinicians in the early detection and management of ICP, ultimately safeguarding maternal and neonatal health.

## Data Availability

The datasets used and analyzed during the current study are available from the first author upon reasonable request.
